# Comparison of Bur Abrasion and CO_2_ Laser in Treatment of Gingival Pigmentation: 6 Months Follow-Up

**DOI:** 10.3290/j.ohpd.b1492771

**Published:** 2021-06-01

**Authors:** Behnaz Roshannia, Maryam Nourelahi, Tahere Ahmadpanahi, Anahita Norouzifard, Shiva Shivaei Kojoori

**Affiliations:** a General Dentist, Dental School, Semnan University of Medical Sciences, Semnan, Iran. Experimental design, perform the experiment, selecting the patients, performing treatment procedures.; b Assistant Professor of Periodontology, Department of Periodontology, Dental School, Semnan University of Medical Sciences, Semnan, Iran. Idea, experimental design, performing treatment procedures, writing the manuscript.; c Graduate, Midwifery Department, Amiralmomenin Hospital, Semnan, Iran. Performed statistical evaluation, patient recruitment.; d Periodontist, Department of Periodontology, Dental School, Semnan University of Medical Sciences, Semnan, Iran. Took standard photographs and analysed them.; e Assistant Professor of Orthodontics, Department of Orthodontics, Dental School, Semnan University of Medical Sciences, Semnan, Iran. Patient recruitment, performed statistical evaluation, analysed the standard photographs.

**Keywords:** laser, bur abrasion, gingival depigmentation, pigmentation, CO_2_

## Abstract

**Purpose:**

Pigmentation of gingiva is an aesthetic problem. Until now, various methods have been introduced for removal of gingival pigmentation. The purpose of this study was to compare bur abrasion and CO_2_ laser methods in removing gingival pigmentation.

**Materials and Methods:**

Twelve patients aged 20–40 years old with the chief complaint of physiologic gingival pigmentation participated in this study. For these patients, gingival depigmentation was performed using two treatment modalities including bur abrasion and CO_2_ laser in a split-mouth design. Gingival depigmentation was performed in the right half of the anterior maxillary and mandibular sextants using bur abrasion method by means of diamond bur on a high-speed headpiece with vigorous water irrigation and the left half of the anterior maxillary and mandibular sextants using a CO_2_ laser. CO_2_ laser was set at: peak power 252 watts, repeat time 20 ms and pulse duration 200 microsecond which was used in a non-contact position. The area of pigmentation was calculated via gridlines in the Microsoft Paint software 1 and 6 months after the procedure. Gingival recession was also determined before, and at 1 and 6 months after the procedure.

**Results:**

The area of gingival pigmentation was not statistically significantly different between the two treatment modalities before the procedure (p = 0.452), 1 month (p = 0.443) and 6 months after the procedure (p = 0.202). There was no statistically significant difference in the variance of pigmented area at different times in the two methods. In both CO_2_ laser and bur abrasion methods, the mean area of pigmentation was statistically significantly different in the follow-up period (p < 0/001), in a way that the change in the area of pigmentation was statistically significant 1 month after treatment (p <0.001) and also 6 months after treatment (p < 0.001) compared to before. The change in the area of pigmentation between 1 and 6 months after treatment was not statistically significant in both CO_2_ laser (p = 0.157) and bur abrasion method (p = 0.150). No increase in gingival recession was observed in any of the patients.

**Conclusion:**

Both treatment modalities can effectively treat gingival pigmentation. No increase in gingival recession was observed. Conventional method and CO_2_ laser were not statistically significantly different during a follow-up period of 1 and 6 months.

From a long time ago, aesthetic has been playing an important role in facial harmony, beautiful smile, sense of self-satisfaction and quality of life. A beautiful smile is not only the result of favourably shaped teeth, but also the result of nice form and colour of the gingiva.^7^

There are some issues compromising gingival aesthetics, such as gingival pigmentations.^34^ Pigmentation is defined as the change in the colour of skin, oral mucosa or gingiva with various aetiologies. It is classified as physiologic and pathologic types.^8^ Oral pigmentation is associated with different intrinsic and extrinsic etiologic factors but it is mainly associated with five primary pigments including melanin, melanoid, oxyhaemoglobin, reduced haemoglobin and carotene.^28, 36^

Gingiva is one of the most frequent sites in the oral cavity which represents pigmentation.^30^ Intrinsic pigmentation is mostly the result of melanin precipitation in epidermal layers. Melanin is a natural pigment which is represented in the oral cavity 3 years after birth. Gingival pigmentation is more frequent in labial and buccal gingiva than lingual and palatal. Pigmentation is also seen more frequently in the anterior site of the oral cavity than the posterior site. Based on recent studies, the frequency of gingival pigmentation is 60% in the gingiva, 22% in the oral mucosa and 15% in the tongue.^11,33^

Melanoblasts are normally present in basal layer of lamina propria. Melanoblasts mostly accumulate in the attached gingiva, then in papilla and marginal gingiva, respectively. Abundance of melanocytes in attached gingiva is 16 times as much as that in the free gingiva.^8,9^

Oral pigmentation is observed in all racial groups and equal in both genders.^8^ Usually there is no difference in number of melanocytes between white and black people, but the difference is the amount of produced melanin and the activity of melanocytes.^28^ The abundance of gingival pigmentation in the Iranian population is 43.47% and the intensity is reported to be intermediate, which is more than European population and less than eastern Asia.^37^

Most of the time gingival pigmentation is physiologic, causes no harm, and is more of an aesthetic problem. It is important to consider aetiology before treatment.^17,39^ Different methods have been introduced for removal of gingival pigmentation such as the surgical blade technique, bur abrasion, free gingival graft, chemical techniques, electro-surgery, cryotherapy and different kinds of lasers including neodymium doped Yttrium–aluminium–Garnet (ND-YAG), Erbium (Er-YAG) lasers, carbon dioxide (CO_2_) and diode lasers.^1,12^ Laser treatment is based on a phenomenon called photothermolysis, which is the conversion of light into heat.^15^ Diode laser beam is absorbed highly by soft tissue and chromophores which is widely used in gingival depigmentation.^5^ CO_2_ laser ablates and vaporises the soft tissue and is a good choice for treatment of gingival pigmentation because it causes less tissue harm compared to Argon or Nd:YAG lasers.^10,11^ Er:YAG laser is frequently used in hard tissue such as bone or tooth according to hydrokinetic system theory but can also be used for gingival depigmentation in special conditions. CO_2_ laser has shown to be equally effective in gingival depigmentation compared to Er:YAG laser.^12,15^

Conventional method for removal of pigmentation usually refers to bur abrasion or scalpel blade techniques. In this method the gingival epithelium is removed using a surgical blade or diamond bur and is healed by means of secondary intention. However, it usually entails bleeding and some discomfort for the patient.^32,35^

Wavelength of CO_2_ laser beam is within the infrared range, which is mainly absorbed by water not melanin. The high water content of oral soft tissues leads to high absorbance of CO_2_ laser beam, which causes depigmentation in a non-selective way.^38^

Laser can also have anti-infective properties which can be considered as an advantage in oral surgery.^3,18^

In this study we aim to compare two treatment modalities including CO_2_ laser and bur abrasion (conventional method) in treatment of gingival pigmentation.

## Materials and Methods

This study is designed as a clinical trial with IRCT number: IRCT20170601034288N1 and is approved in the ethical committee of Semnan University of Medical Sciences. In this study, 18- to 40-year-old patients with the chief complaint of physiologic gingival pigmentation in anterior sextants of the maxilla and mandible, who were referred to Semnan University of Medical Sciences dental clinic, were evaluated.

The inclusion criteria were as follows: physiologic entity of pigmentation; medium to severe pigmentation based on the Dummett-Gupa index index^29^; plaque index less than 20%; no medical conditions disturbing tissue healing.

Exclusion criteria included: background or environmental factors as the aetiology of gingival pigmentation such as endocrine diseases, blood dyscrasia or congenital syndromes; smoking or taking some drugs which induce gingival pigmentation; pregnancy or breastfeeding; thin biotype of periodontium; acute gingival or periodontal diseases.

Based on these criteria, 12 patients participated in the study and consent forms were filled out by all the patients. First they were educated on how to reduce dental plaque accumulation. Patients underwent professional scaling and educated on how to control plaque accumulation 1 week before the start of the study. Then they were treated for gingival pigmentation with a split-mouth design using the two treatment modalities including bur abrasion and CO_2_ laser methods. The right half of the anterior sextants of maxilla and mandible including right central, lateral and canine were treated with the bur abrasion method. The left half of anterior sextants of maxilla and mandible including left central, lateral and canine were treated with CO_2_ laser.

In the conventional method, local anaesthesia including 2% lidocaine and epinephrine 1/100,000 (Xylopen, Iran) was applied. Pigmented epithelium was abraded with round diamond bur (Yun DA, China) connected to a high-speed headpiece (Easyinsmile CEX, Taiwan) with feather-like brushing motions and vigorous irrigation by normal saline. Pressure with moist gauze was applied and then the area was covered with a periodontal pack.

In the laser site, topical lidocaine was sprayed on the site of procedure and local infiltration anaesthesia was only used for patients who sensed some kind of discomfort. Protective glasses were worn by the practitioner and the patient for safety reasons. CO_2_ laser (Daeshin Enterprise, Korea) was set at: peak power 252 watts, repeat time 20 ms and pulse duration 200 microsecond which was used in a non-contact position. The beam spot size was 100 µm. Care was taken not to harm dental and other surrounding structures during the procedure.

All the procedures were done by the same periodontist.

**Fig 1:**
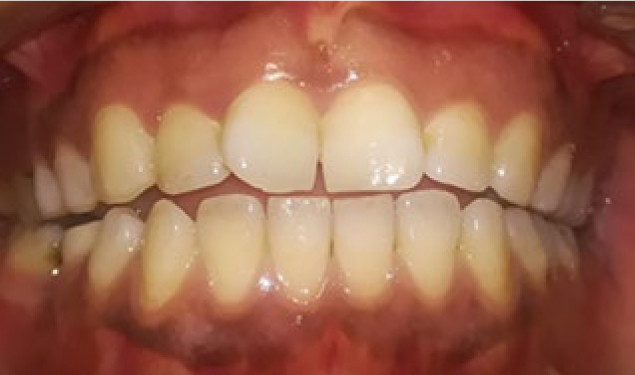
Standard photograph taken before depigmentation procedures.

**Fig 2:**
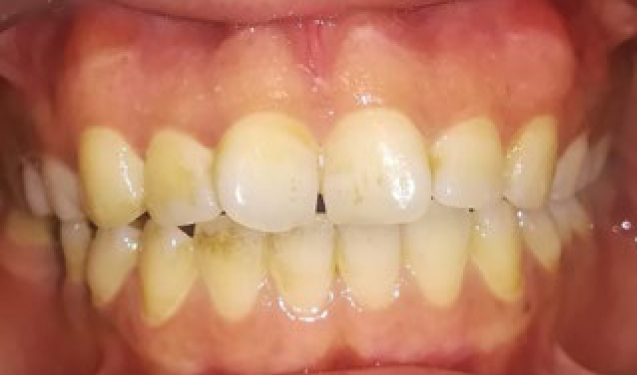
Standard photograph taken 1 month after depigmentation procedures.

**Fig 3:**
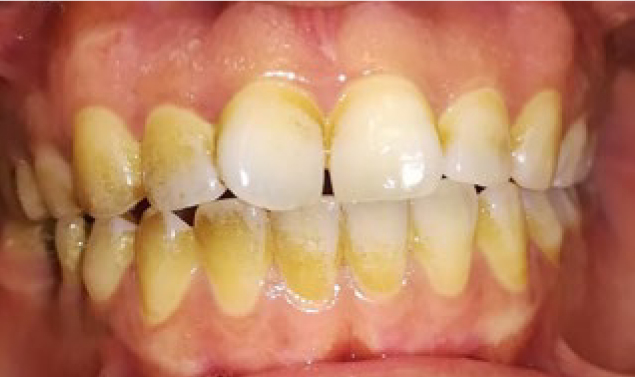
Standard photograph taken 6 months after depigmentation procedures.

Chlorhexidine 0.2% was prescribed to the patients twice a day for 1 week and they were advised on not brushing for 3 days and use a soft brush for plaque control after that. The patients were also asked to avoid spicy food for several days. To compare the results, standard photographs were taken before the procedure and 1 and 6 months after the procedure. Position of the patients were similarly up right for all the patients, distance to the camera was 30 cm from lens of the camera to each patient’s nasion. Light of the operation room was also similar for all the patients. A digital camera (Canon, Japan) with 10 MP resolution and flash off was used. Photographs were analysed using Microsoft Paint software version 6.1. Figures 1 to 3 are provided as an example of intraoral photographs taken of a patient before depigmentation procedures, and at 1 and 6 months after that. The area of pigmentation was determined and compared by counting standard 1 mm^2^ grids in the Microsoft Paint before, 1 and 6 months after the procedure.

Gingival recession was also determined before, 1 and 6 months after the procedure. To do so, the distance between gingival margin and Cemento Enamel Junction was evaluated before, 1 and 6 months after the procedure.

Data was analysed using Shapiro–Wilk, Paired t-test, Wilcoxon test, repeated measures ANOVA and Bonferroni post-hoc test. SPSS version 23.0 was used and statistical significance level of 0.05 was considered.

## Results

Twelve patients with chief complaint of gingival pigmentation within the age range of 20–37 years were participated in this study. The average age of the patients was 28.5 ± 5.7 with median of 27.5. The minimum age was 20 and the maximum age was 37. The age distribution of the patients are shown in Table 1.

**Table 1:** Age distribution of the patients

Percentage	Number	Age (y)
25/0	3	<25
33/3	4	25–29
41/7	5	≥30
100	12	Total

Average, standard error, median and interquartile range of the area of pigmentation before the procedure, one and six months after the procedure are summarised in Table 2. The area of pigmentation was not significantly different between the two treatment modalities before the procedure (p = 0.452), 1 month (p = 0.443) and 6 months (p = 0.202) after the procedure. The change in the area of pigmentation was not also significantly different between two treatment modalities in different timelines, which is found in Table 2. The results of repeated measures analysis of variance (ANOVA) did not show any interrelationship between the treatment modality and the time (p = 0.773) In the CO_2_ laser group, the average area of pigmentation was significantly different in the three time lines (p < 0.001). The decrease in pigmentation 1 month after the treatment compared to the before (p < 0.001), and 6 months after the treatment compared to the before (p <0.001) was significantly different. But the change between 1 and 6 months in the two groups was not significantly different (p = 0.157).

**Table 2:** Statistical parameters of the area of pigmentation before the procedure, one and six months after the procedure

	Treatment modality								P value
	CO_2_ laser				Conventional				
	Interquartile range	Median	Standard error	Average	Interquartile range	Median	Standard error	Average	
Before treatment	44.0	46.5	8.0	52.2	44.0	51.0	7.7	51.2	0.452
One month after treatment	8.00	1.0	1.2	4.6	5.00	5.50	1.7	6.7	0.443
Six months after treatment	8.00	5.5	2.8	6.7	10.00	7.00	1.7	8.8	0.2.2
The change between before and 1 month	47.5	38.5	8.0	47.7	38.2	45.5	6.9	44.5	0.263
The change between before and 6 months	47.5	38.5	8.0	45.6	33.7	42.5	6.9	42.3	0.288
The change between 1 and 6 months	4.7	0.00	1.0	1.2	5.50	0.00	1.0	2.2	0.854

Also in the conventional group, the average area of pigmentation was significantly different in the three time lines in a way that decline in the area of pigmentation 1 month after treatment (p < 0.001) and 6 months after treatment (p < 0.001) was significantly different compared to the before but the change between 1 and 6 months after treatment were not significantly different (p = 0.150).

No gingival recession was observed in the 1 and 6 months follow-up in any of the patients.

## Discussion

So far, no single best method has been introduced for gingival depigmentation and the selection is mainly based on each individual’s preference and clinical experience.

Bur abrasion is a simple, cheap and effective method for gingival depigmentation but it causes unpleasant bleeding during and after the procedure and has the potential to harm bone surface during abrasion with rotary instruments.^13, 22^ Electro-surgery has some disadvantages like heat accumulation and tissue destruction and technique sensitivity. Cryosurgery is reported to cause tissue inflammation and destruction.^28^ Laser is a good method which is able to remove a thin layer of epithelium. Bleeding does not occur during laser application and laser wound is sterile and has no inflammatory reaction. The wound of laser does not usually cause the pain that is reported by electro surgery.^28^

In the present study, we found out that bur abrasion and CO_2_ laser are equally effective in removing gingival pigmentations. We used the Daeshin Enterprise CO_2_ laser because it costs less than most other laser equipment and is frequently available in the dental universities of Iran. Our results are similar to Rhini Negi et al’s who compared a soft tissue trimming bur technique with diode laser (0.5–1.5 W continuous wave) in a split-mouth design. They used DOPI (Dummet oral pigmentation index) and GPI (gingival pigmentation index) to evaluate the area of pigmentation before and after treatment. They noticed no statistically significant difference between the two methods. Recurrence of pigmentation occurred in a few patients.^27^

The reasons for recurrence of pigmentation is not fully understood. Migration of melanocytes from adjacent areas to the areas under treatment could be a reason for recurrence of pigmentation in the areas previously treated but usually with less intensity. Another possible aetiology could be activation of melanocytes during treatment. These melanocytes commence synthesis of melanin, which has been observed in treatment with laser.^27^

Deepak also found no statistically significant difference in the efficiency of gingival depigmentation between the three methods of bur abrasion, electro-surgery and surgical blade.^31^

The results of the present study are a bit different from those in the study of Ameet Mani et al, who compared diode laser, bur abrasion and surgical blade techniques and found diode laser to be more effective. They used the melanin pigmentation index (MPI) before and after treatment and punch biopsy for histologic evaluation. They reported that wound healing after laser therapy requires more time than bur and surgical techniques. The sample was only one person.^22^ Bur abrasion, electro-surgery and scalpel blade techniques had no statistically significant difference in the results of gingival depigmentation in the study conducted by Kathariya et al. They observed no difference in the three techniques regarding recurrence of pigmentation but some discomfort was reported by the patients treated with electro-surgery. In their review article, they stated that laser has the advantages of both conventional and electro-surgery techniques including rapidity and minimal bleeding.^13^

Er:YAG laser and bur abrasion were both effective methods after 4 weeks of treatment, which is observed by Kwang-Myung Lee et al. However, Er:YAG laser (250 mJ, 15 Hz) caused some bone dehiscence in this study and was not considered convenient in patients with a thin biotype of periodontium.^19^ After a follow-up period of 3 months, no statistically significant difference was observed in the study conducted by Murthy et al between rotary abrasion, surgical blade and diode laser methods regarding wound healing and recurrence rate but delayed healing occurred with diode laser compared to conventional methods.^24^ No recurrence of pigmentation was observed in the three patients treated with the bur abrasion technique after 18 months in the study conducted by Sameer et al.^23^

CO_2_ laser was suggested as an effective and advanced method in the treatment of gingival pigmentation by Ozbayarak et al.^28^ Hegazy et al used CO_2_ laser for depigmentation in one patient and no recurrence was found after 6 months follow-up. They suggested that less pain is induced with CO_2_ laser compared to the conventional methods.^11^

In another study by Hegde et al, surgical stripping, CO_2_ laser and Er:YAG were compared. After 6 months follow-up, they observed less recurrent pigmentation in the surgical stripping method followed by CO_2_ laser. In this study Er:YAG laser showed more recurrent pigmentation which could be explained by the fact that it is highly absorbed by water and forms a very thin surface interaction layer and less tissue degeneration. This phenomenon, which is called water-mediated explosive ablation, allows ablation without any scarring but also increases the probability of recurrence. This is a reason why we chose CO_2_ laser over Er:YAG in the present study. CO_2_ laser causes cell rupture due to a rise in intracellular temperature, which seals blood vessels in the surrounding tissue and delays cell migration leading to the less recurrence rate.^12^

The absence of thermal effect in the conventional surgery method makes it easier to be used in deep pigmentations. This method also causes a larger layer of cell death that may delay cell migration and lowers recurrence rate. A disadvantage of conventional method is that it was reported to cause more discomfort for the patients compared to the laser, which is attributed either to the protein coagulum formed on the wound surface or sealing nerve ends by laser.^12^

Esen et al treated 10 patients for gingival pigmentation with CO_2_ (10 watts, 0.8 mm spot size, 20 Hz, 10 milliseconds) laser and reported it to be a very effective method. The follow-up period was 2 years. A photo analysis software was used to determine area of pigmentation. Two cases of partial repigmentation was observed, which was partly related to smoking habit.^10^ In Negpal’s case report, using four different methods for gingival depigmentation including bur abrasion, scalpel scraping, diode laser and cryotherapy, it was concluded that all the methods are equally effective in removing gingival depigmentation. Each of them had some advantages and disadvantages though, such as the cost of the laser, and bleeding and pain in scalpel and bur abrasion.^25^

It is very important to choose patients with similar systemic, local and environmental conditions to avoid bias in interpretation of therapeutic results. In the present study, patients were similar regarding aetiology of pigmentation, systemic conditions, medical history and age range.

Atsawasuwan et al reported successful results in treatment of gingival pigmentation with Nd:YAG laser but gingival fenestration was observed in some patients.^4^ In a study by Kishore et al about CO_2_ and Nd:YAG laser, both lasers were optimum for treatment of gingival pigmentation and no gingival recession or inflammation was observed after treatment.^16^

One expected result of CO_2_ laser is that it implies less risk of destruction of periosteum compared to other kinds of laser such as argon and Nd:YAG. The depth of tissue destruction caused by CO_2_ laser is 50–100 micrometres which is less compared to Nd:YAG and Argon lasers (600 and 200 micrometres, respectively).^10,26^ In the present study, no gingival or bone fenestration was observed after the follow-up period.

The time of follow-up is chosen based on different parameters. Severity of pigmentation and racial properties is one example. More severity of pigmentation and more rate of repigmentation has been reported in dark skinned people.^20^ In different studies, time of follow-up has been in the range of 1 month to 2 years, but 6 to 9 months is mostly determined as the optimum time for follow-up period.^2,6,14,20^ However, it is stated that within 1.5 to 3 years, repigmentation may return to its clinical baseline.^21^ We followed our patients for 6 months. It seems that a longer time to follow the patients would be better.

## Conclusion

In the present study, no gingival recession occurred in the sites of the two treatment modalities. The results are confirmed by a few studies which evaluated the effects of different methods of gingival depigmentation on gingival recession including surgical abrasion, Er:YAG laser and CO_2_ laser.^10,23,34^

But in the study by Atsawasume et al, one out of four patients showed gingival recession after treatment with Nd:YAG laser. Marginal gingiva returned to normal after 9 months. The author suggested that in thin gingival tissue or the gingiva around root prominence, pigmentation should be removed gently.^4^
